# Non-genetic diagnostic investigations in monogenic Ehlers-Danlos syndromes

**DOI:** 10.1515/medgen-2024-2062

**Published:** 2024-12-03

**Authors:** Fleur S. van Dijk, Chloe Angwin, Neeti Ghali, Johannes Zschocke, Bart Wagner

**Affiliations:** London North West University Health Care NHS Trust National EDS service, London North West University Health Care, NHS Trust Watford Road HA1 3UJ Harrow United Kingdom; London North West University Health Care NHS Trust National EDS service Watford Road HA1 3UJ Harrow United Kingdom; Imperial College London Department of Metabolism, Digestion and SW7 2AZ London United Kingdom; Medical University Innsbruck Institute of Human Genetics, Department of Genetics Peter-Mayr-Str. 1 6020 Innsbruck Austria; Royal Hallamshire Hospital Electron microscopy section, Histopathology Department Glossop Road S10 2JF Sheffield United Kingdom

**Keywords:** Ehlers-Danlos syndromes, Connective Tissue, Extracellular matrix, Collagen, Electron Microscopy

## Abstract

With increased application of Next Generation Sequencing (NGS) in the diagnosis of monogenic Ehlers-Danlos syndromes, there is an increased probability to identify variants of unknown significance. Additionally, in some cases no genetic alteration may be identified whilst there is a strong clinical suspicion on a monogenic EDS type. The diagnostic value of non-genetic investigations, which prior to NGS were quite commonly used to support the clinical diagnosis of monogenic EDS types, is explored. In addition, new structural/functional investigations that could deliver evidence towards pathogenicity are discussed. It appears that certain functional and/or structural investigations used frequently in the past can remain helpful and can provide additional evidence that may confirm a clinical diagnosis of a monogenic EDS type. However, there is a need for the development of novel structural/functional studies for monogenic types of EDS. The level of evidence of such studies for application in the established diagnostic DNA variant classification criteria remains to be determined.

## 1 Introduction

EDS (Ehlers-Danlos syndromes) is an umbrella term encompassing 13 different EDS types. Whilst hypermobile EDS is currently diagnosed based on strict clinical criteria, the other 12 EDS types are rare, proven monogenic connective tissue conditions that share clinical features of joint hypermobility, skin hyperextensibility and/or fragility, and generalised tissue fragility to a variable degree.

Genetic causes of EDS types can be grouped in (a) genes encoding collagen type I, III, V and XII, (b) enzymes involved in their biosynthesis or (c)enzymes involved in proteoglycan biosynthesis [1]. Gene panel testing with massively parallel sequencing (Next Generation Sequencing, NGS) is currently the gold standard to confirm a diagnosis of a monogenic EDS type.

Gene variants are currently assessed according to the American College of Medical Genetis (ACMG) criteria [2]. However, there are still cases where there is a clinical diagnosis of a monogenic EDS type, and no genetic cause is identified or where a variant of unknown significance (VUS) is reported, and more evidence is needed to upgrade the variant to (likely) pathogenic.

Here we explore whether in these scenarios non-genetic investigations, which prior to NGS were quite commonly used to support the clinical diagnosis of monogenic EDS types, remain diagnostically valuable. We discuss in more detail (i) electron microscopy of dermal tissue, (ii) collagen electrophoresis in fibroblasts, and lastly (iii) urinary **H**igh **P**erformance **L**iquid **C**hromatography cross-linking assay and examine whether and in what capacity these could be used to aid the diagnostic confirmation of a monogenic EDS type. In addition, we briefly discuss new functional analyses that have been published for specific EDS types.

## 2 Transmission Electron Microscopy in monogenic Ehlers-Danlos types

Transmission Electron Microscopy (TEM) of a 3–4 mm skin biopsy has been used to support the clinical diagnosis of monogenic Ehlers-Danlos types prior to and in combination with DNA analysis. In the past, several findings on TEM had been reported to be indicative of a monogenic EDS type namely (i) duplication of lamina densa; (ii) collagen flowers (**figure 2b**), (iii) variable diameter of collagen fibrils (**figure 2a**); (iv) disorganisation, distortion or kinking of collagen fibres (**figure 3a and 5**) and (iv) of other dermal structures including elastic fibres and fibroblasts (**figure 3b**). Importantly, Angwin et al. [3] could not confirm that duplication of the lamina densa was specifically associated with vascular EDS as was reported previously [4]. Musculocontractural EDS caused by recessive *CHST14* or *DSE* variants is a relatively more recently identified monogenic EDS type. Hirose et al. report significant differences in skin TEM of individuals with mcEDS-*CHST14* using cupromeronic blue staining to visualise glycosaminoglycan (GAC) chains showing important differences between affected individuals and healthy controls [5]. This is an example of a structural study that could contribute as evidence towards a diagnosis of *CHST14* mcEDS.

**Figure 1:**
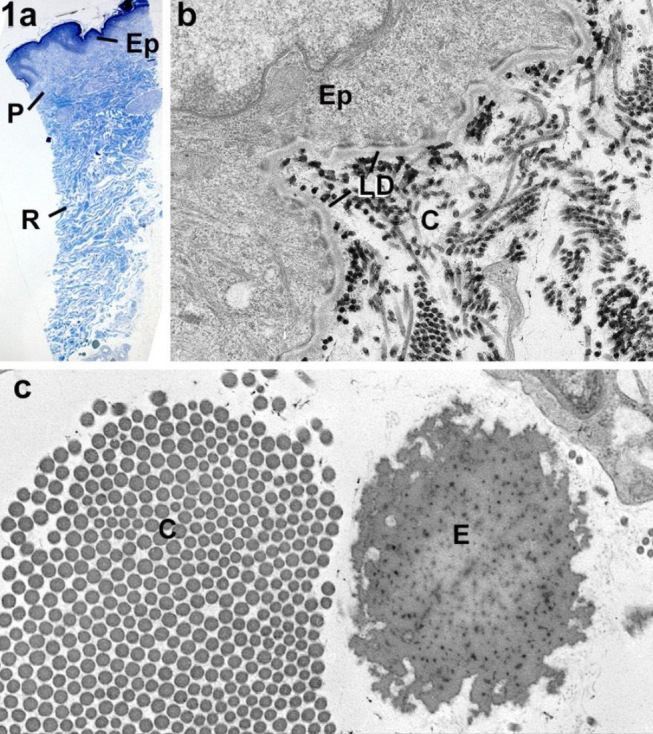
Normal skin, images reproduced with permission from Angwin et al. (3) a. Light microscopy of a longitudinal section through a skin biopsy showing the surface epidermis (Ep) plus the papillary (P) and reticular (R) dermis. b. Transmission electron microscopy (TEM) showing the junction between the epidermis (Ep) and dermis with collagen fibrils (C) separated the single layer of the lamina dense (LD). c. Section from the reticular dermis show a cross section through part of collagen fibre consisting of collagen fibrils with a circular outline (C) and solid elastic fibre (E).

Collagen flowers are a generally consistent finding in individuals with a definitive diagnosis of classical EDS even though there are exceptions, and the amount and size of collagen flowers differ between individuals with cEDS [5]. Importantly, collagen flowers can also be seen in other monogenic EDS types as well as related monogenic connective tissue conditions including Osteogenesis Imperfecta (OI) [6] and Ullrich muscular dystrophy. They have also been reported in individuals with a clinical diagnosis of hEDS [4] prior to publication of the hEDS clinical criteria in 2017. This suggests that collagen flowers, though almost invariably present in individuals with cEDS, are not specific for cEDS, monogenic EDS, and potentially monogenic disease.

**Figure 2:**
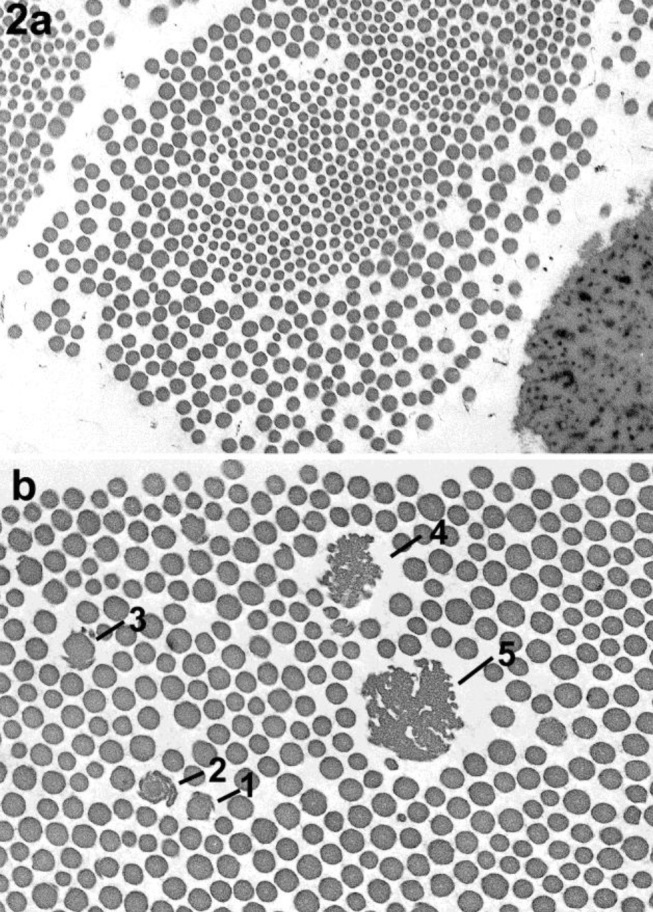
Collagen fibre cross section, images reproduced with permission from Angwin et al. (3) a. Cross section through a collagen fibre demonstrating marked variation in the diameter of the collagen fibrils. b. Cross section illustrating the appearance of collagen flowers. Note the apparent progression in complexity of the abnormal fibrils (1 to 5).

Variable diameter of collagen fibrils has been reported in vascular EDS (vEDS) [7–9] and classical EDS (cEDS) [10–13] and is thought to indicate disruption to the processing and organization of collagen. A recent study confirmed a higher variation of collagen fibril diameters (high coefficient of variation, COV) due to presence of many small fibrils in individuals with genetically confirmed vEDS, compared to controls [14]. Intriguingly, comparison of skin TEM between infants, individuals with vEDS, and adult controls, revealed a similar COV in infants and vEDS individuals, significantly higher than in controls. It was concluded that a high COV resulting from the production of many small collagen fibrils may be physiological in infants, whereas in vEDS it reflects unstable retention of collagen bundles due to ER stress [15]. A study that examined 13 patients with *PLOD1* related kyphoscoliotic Ehlers Danlos syndrome (kEDS) found variable diameter of fibrils along with abnormal collagen fibre outline compared with a control group [16]. Variable diameter of collagen fibrils has also been documented in several other disorders including osteogenesis imperfecta [6].

**Figure 3:**
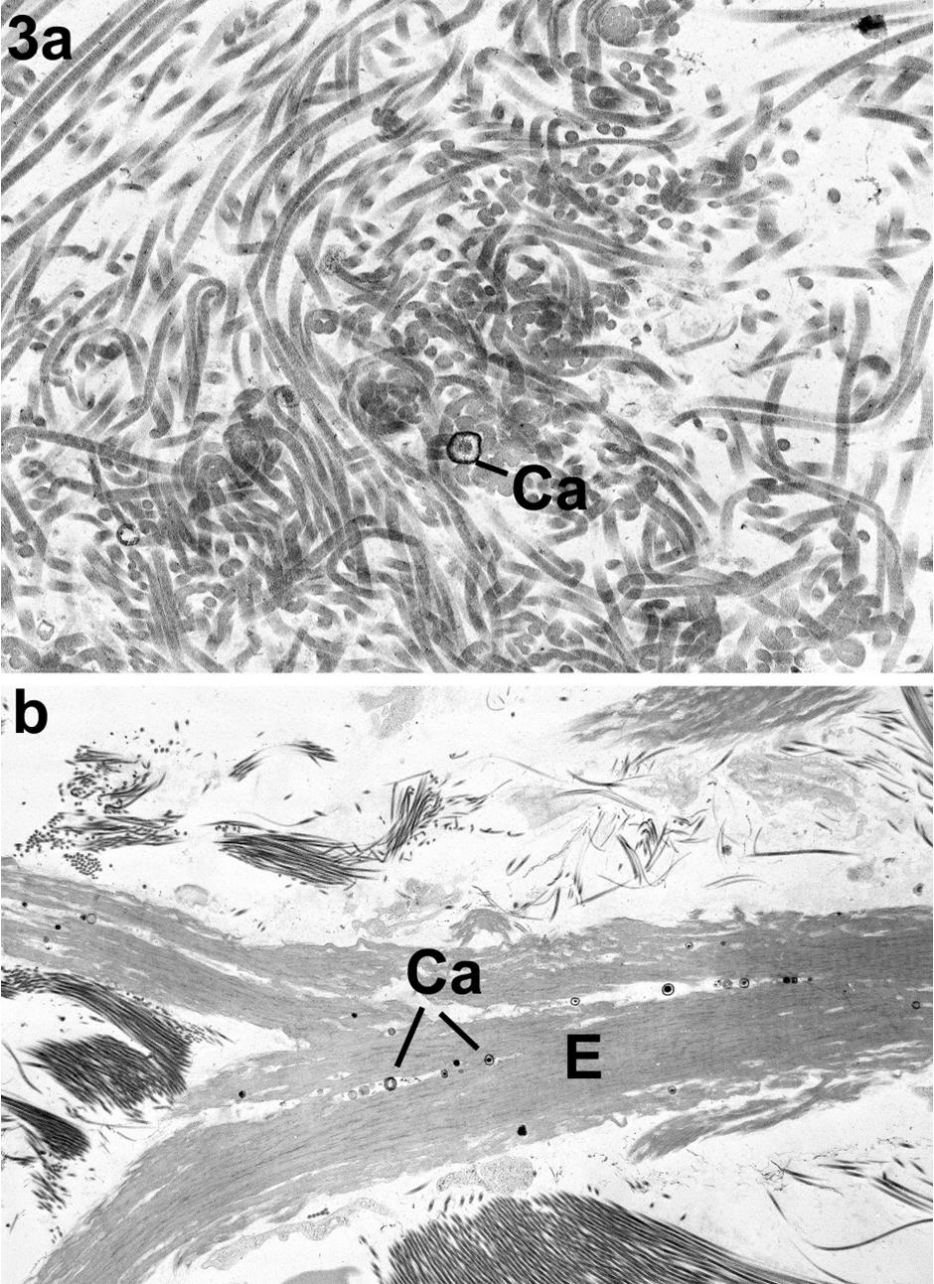
Disorganisation and kinking images reproduced with permission from Angwin et al. (3) a. Sharp kinking and intertwining of collagen fibrils with a small calcium deposit (Ca). b. Longitudinal section through an elastic fibre (E) showing branching and the presence small calcium deposits (Ca).

**Figure 4:**
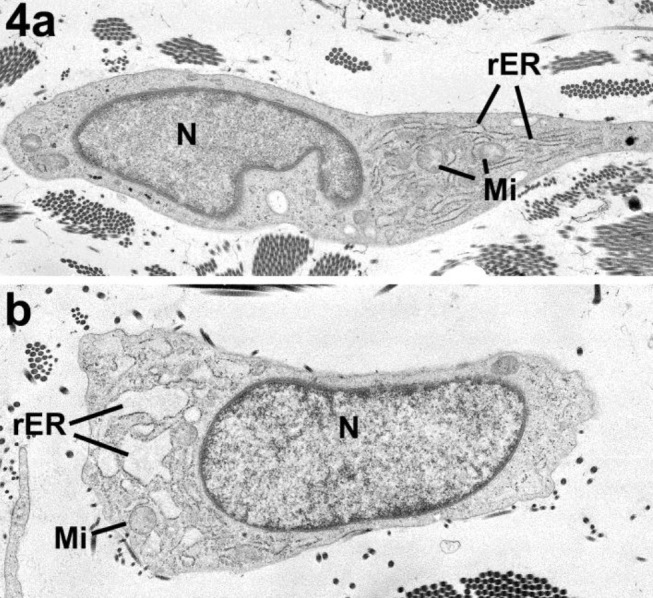
Rough endoplasmic reticulum changes, images reproduced with permission from Angwin et al. (3) a. Normal fibroblast: (N) – nucleus, (Mi) – mitochondria within cytoplasm, (rER) – rough endoplasmic reticulum with narrow lumen. b. Abnormal fibroblast: (N) – nucleus, (Mi) – mitochondria within cytoplasm, (rER) – rough endoplasmic reticulum with marked dilation.

**Figure 5:**
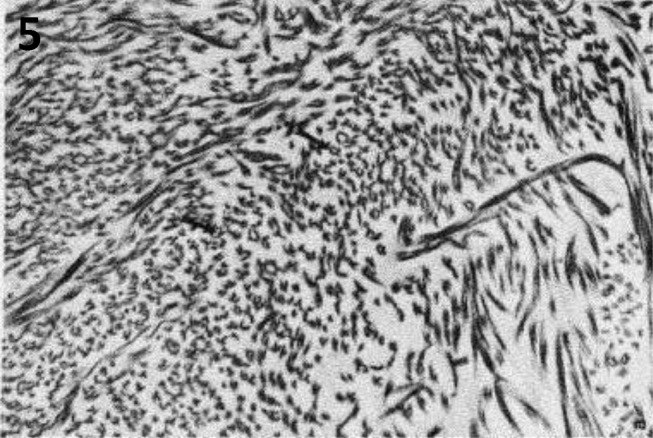
Hieroglyphic fibrils pathognomonic for dermatosparaxis EDS, images reproduced with permission from Reardon et al. **(17)**

Disorganisation, distortion, or kinking of collagen fibres has been reported in dermatosparaxis EDS (dEDS), [17] with a pathognomonic appearance of collagen fibres that look like “hieroglyphs” on TEM in this condition (Figure 5). However, van Damme et al [18] reported that in four patients with dEDS who had skin biopsies, EM abnormalities did not represent the pathognomonic hieroglyphic pattern. In two of these four patients with dEDS, the pattern resembled of that seen in arthrochalasia EDS due to specific *COL1A1* and *COL1A2* variants. Therefore, presence of the hieroglyphic pattern strongly supports a diagnosis of dEDS and may be used as evidence for VUS interpretation, whereas absence does not exclude this diagnosis. Importantly, TEM abnormalities showing very irregular contours and a branched appearance with irregular interfibrillar spaces but with some preservation of cylindrical shape can also be seen in individuals with aEDS due to specific *COL1A1* and *COL1A2* variants resulting in retention of the N-propeptide in the mature α1(I) and α2(I) molecules and leading to impaired fibril formation. Comparable TEM findings in individuals with dEDS and aEDS due to specific *COL1A1* and *COL1A2* variants are understandable as dEDS is due to deficient activity of ADAMTS-2, an endopeptidase that excises the N-propeptide of procollagen chains [19, 20]. Redman et al. [9] concluded that dilated rough Endoplasmic Reticulum (rER) visible on TEM should be considered an abnormal finding (figure 4b). It has been proposed that dilated rER is a sign of the unfolded protein response (UPR). This is a pathway triggered by ER stress due to abnormal protein folding and secretion, potentially preventing the misfolded protein from being transferred to the proteosome for degradation. The authors argued that dilated rER could provide additional evidence for a diagnosis of vEDS or a related inherited connective tissue condition including severe types of OI. However, recent literature indicates that when protein aggregates in the ER are too large, another cellular mechanism named ER-phagy is activated to promote the removal of aberrant proteins and preserve ER integrity [21]. Therefore, absence of dilated rER would not exclude a diagnosis of vEDS or related inherited connective tissue conditions. In addition, Ishikawa et al. identified rER dilatation in individuals with a high COV but the dilatation was not as marked as observed by Redman et al. In the low COV group only mild or non-dilated rEDS were identified [14].

**Figure 6A:**
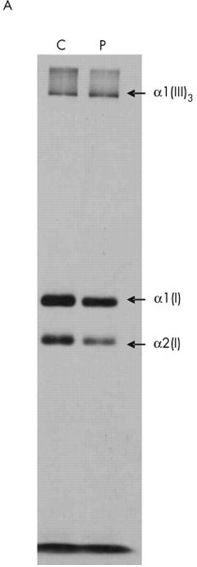
SDS-PAGE collagen electrophoresis, images reproduced with permission from Symoens et al. **(32)** Collagen electrophoresis in an individual with an OI-EDS overlap phenotype due to a heterozygous c.3790G>A variant in *COL1A1*. This alteration caused alternative splicing and generated two different mutant transcripts p. ([Met1264Val; Met1264Glufs*59]). It was predicted that the second mutant transcript would lead to truncated pro-alpha1(I) collagen protein and functional haploinsufficiency. This is shown in figure 6A as marked reduction in the intensity of the collagen type I molecules secreted in the medium (P) compared to a control sample (C) **(32).** The arrows indicate the migration positions of type I collagen α1(I) and α2(I) chains and type III collagen (α1(III)3 which show no abnormalities **(32).**

Angwin et al. reported that no TEM findings were specifically associated with an EDS type, but there was a higher percentage of abnormal skin biopsy findings in individuals with (likely) pathogenic variant(s) and a definitive diagnosis of a monogenic EDS type [4]. TEM findings as outlined above do not seem to be specific for a type of EDS (apart from dEDS or aEDS) and have also been identified in other conditions including other inherited connective tissue conditions. In case of a clinical diagnosis of classical EDS and an identified VUS in *COL5A1*/*COL5A2* or an unidentified genetic cause, the presence of collagen flowers on TEM can potentially be supporting evidence that may contribute to the upgrade of a VUS. Indeed, in combination with results from other genetic and non-genetic investigations, it can also signpost to a missed genetic defect in *COL5A1/2* as suggested by Colman et al [22].

Currently, it appears that specific TEM abnormalities are pathognomonic only for dEDS and aEDS. Presence of abundant collagen flowers on TEM could have added value as an additional piece of evidence in case of a *COL5A1*/*2* VUS (presence of abundant collagen flowers). TEM abnormalities including collagen flowers but also variable diameter of collagen fibrils, disorganisation, distortion or kinking of collagen fibres and dilated rER could, together with clinical features serve as a marker of an inherited connective tissue condition requiring further genetic investigations. However, it would be important to discuss whether, when and how specific TEM findings could be considered in the ACMG criteria, and this will likely require discussion in expert workgroups. This process would be facilitated if data regarding the specificity and sensitivity of TEM to contribute to a diagnosis of a monogenic EDS type would be available. To generate these data, it would be helpful to compare TEM in cohorts of individuals with different types of molecularly confirmed EDS compared with healthy controls. In addition, standardizing TEM assessments as much as possible would be helpful to reduce observer bias.

## 3 Protein analysis with collagen electrophoresis in cardiac-valvular EDS, vascular EDS and classical EDS

Structural variants in genes encoding collagens type I, III, V and XII can cause monogenic types of EDS. Collagen type I, III, and V, belong to the group of fibrillar collagens, which means that they can form fibrils that provide tensile strength, maintain the stability of tissues, and preserve structural integrity [23]. Biochemical testing for monogenic types of EDS caused by structural variants in *COL1A1/2*, *COL5A1/2* or *COL3A1* was widely used as a diagnostic technique before NGS became available. It requires availability of cultured dermal fibroblasts the most accessible connective tissue cells that abundantly express collagen I, III and V.

Previously, proteins synthesized by fibroblasts and other cells were biosynthetically marked with radiolabelled proline (collagen has a much higher proline content than other proteins) and separated based on their molecular weight by sodium dodecyl-sulfate polyacrylamide gel electrophoresis (SDS-PAGE) [18]. This enabled the assessment of the amount of procollagen type synthesized, the quantity secreted into the medium, and the electrophoretic mobility of the different collagen chains [24, 25]. Experience with SDS-PAGE collagen electrophoresis is detailed below. Unfortunately, this specific method with radiolabelled proline is not offered anymore in a diagnostic setting because this requires autoradiographic films that are no longer available. However, fluorescent Cy5-labelling of lysine followed by collagen electrophoresis has been developed as an alternative for the analysis of Collagen type I and Collagen type III [26]. For example, this approach was used in a research setting to elucidate the pathogenic mechanism of a newly discovered genetic cause of OI [27]. Collagen electrophoresis remains a potentially useful technique for the characterisation of monogenic EDS involving collagens type I and III in particular, although currently it is not easily available in a diagnostic setting.

### Collagen type I (including COL1-related OI/EDS overlap disorder, cardiac valvular EDS, vascular EDS,)

Collagen type I makes up approximately 80 % of all collagens in the human body and consists of 2 proα1 and 1 proα2 chains with each proα-chain encompassing a triple helical domain consisting of Gly-X-Y repeats flanked by N and C propeptides. X and Y can be any amino-acid but most frequently are proline and 4-hydroxyproline. The α1 chain is encoded by *COL1A1,* and the α2 chain is encoded by *COL1A2*. The proα chains are synthesized by the rER and then aligned and subequently fold as a triple helix in a zipper-like way from the C-N terminal end. Recent literature reports that the C- and N-propeptides are in fact cleaved intracellularly by specific enzymes, with the N-propetide being transported with collagen type I to the extracellular matrix (ECM)[25]. Subsequently, cross-linking of collagen type I molecules leads to formation of fibrils [23].

Heterozygous null (loss-of-function, LoF) variants in *COL1A1* lead to a 50 % decrease of COL1A1 which typically causes OI type 1 or mild non-deforming OI, representing haploinsufficiency. No disease phenotype is associated with heterozygous *COL1A2* LoF variants, but homozygous LoF variants cause cardiac-valvular EDS [28]. Heterozygous *COL1A1*/*2* variants leading to triple helical glycine substitutions have a dominant negative effect and usually cause OI types 2–4 depending on the gene involved and the type and position of amino acid change [29]. It is noted in general that variants towards the N-terminus may have a milder effect than variants located towards the C-terminus as this is where the folding process starts, and this has been reported for collagen type III as well [30]. Variants at the N-terminus have been identified in several cases of combined OI/EDS overlap syndrome type 1 and 2 or COL1-related overlap disorder [31].

A marked reduction of collagen 1 caused by *COL1A1* LoF variants is usually recognizable on SDS-PAGE [32]. Abnormal alpha1 or alpha2 chains caused by structural variants in *COL1A1* or *COL1A2,* and biallelic variants in genes encoding for proteins involved in (pro)collagen type I post-translational modification, may also give rise to abnormal migration patterns on electrophoresis. This reflects the disturbed intertwining of the three chains forming a triple helix leading to *over*modification of the chains, e. g. hydroxylation or glycosylation that increases molecular weight. Rarely, an abnormal electrophoretic pattern for type I collagen is detected because of an arginine-to cysteine substitution in the *COL1A1* gene coding for the proα1 chain of type I collagen [33]. Specific *COL1A1* arginine-to cysteine substitutions have been reported to cause vascular EDS [34].

### Collagen type III (vascular EDS)

Collagen type III is a homotrimer consisting of 3 identical α1 chains again with C and N-propeptides and a triple helical domain consisting of Gly-X-Y repeats. Triple helical glycine substitutions and splice site variants typically have a dominant negative effect leading to a significantly decreased production of normal (pro)collagen type III molecules (87.5 %). Reduced production (50 %) with clinical manifestation may also be caused by *COL3A1* LoF variants, reflecting haploinsufficiency of the gene. Analysis of type III procollagen synthesized by cultured cells can show abnormalities in synthesis and mobility of type III collagen chains. Alterations in mobility due to slow folding and increased post-translational modification may result from substitutions for glycines in the triple helical domain, as well as from deletions/duplications or splice site alterations. To confirm a diagnosis of vascular EDS, collagen electrophoresis is now often only used to assess outcome of splice-site alterations identified by DNA analysis [24]. As early as 1994 [30] it was reported that, whilst SDS-PAGE electrophoresis can show abnormalities in individuals with vascular EDS, a normal collagen protein profile on electrophoresis analysis does **not** exclude vascular EDS.

### Collagen type V (classical EDS)

Collagen type V has three molecular isoforms: (i) two α1 chains plus one α2 chain, (ii) 3 α1 chains, or (iii) one α1 chain plus one α2 chain and one α3 chain. The different α1, α2 and α3 chains are respectively encoded by *COL5A1*, *COL5A2*, and *COL5A3*. Collagen type V has a quantitatively minor but wide tissue distribution. LOF variants in both *COL5A1* and *COL5A2* lead to a clinical phenotype of classical EDS, reflecting haploinsufficiency. It is important to note that as type V procollagen molecules do not form with more than one proα2(V)‐chain, a 50 % reduction of proα1(V)‐chains results in half-normal amount of collagen type V [22]. Type V collagen is synthesized at low levels by fibroblasts, and alterations in electrophoretic mobility are poorly reproducible, making this approach ineffective for routine diagnostic evaluation [33].

Colman et al. 2021 suggested that in the case of a VUS in *COL5A1* or *COL5A2,* further studies depend on the nature of the VUS and may include (i) mRNA/cDNA studies, (ii) protein studies such as Western blotting and immunofluorescence staining, and (ii) TEM. In case of a clinical diagnosis of classical EDS without identified genetic cause (including normal CNV and transcript studies), the advice is to consider urine analysis (see paragraph 4), TEM (see paragraph 2), and SDS PAGE collagen electrophoresis [22].

## 4 Urinary cross-linking assay (*PLOD1*-related kyphoscoliotic EDS)

*PLOD1* kyphoscoliotic EDS is an autosomal recessive condition characterised by congenital muscle hypotonia, congenital or early onset kyphoscoliosis (progressive or nonprogressive), and generalized joint hypermobility with dislocations/subluxations. It results from a deficiency of the enzyme lysyl hydroxylase 1 (LH1) encoded by *PLOD1*. LH1 hydroxylates lysyl residues on newly synthesized intracellular collagen peptides. After extracellular secretion, specific lysyl and hydroxylysyl residues on adjacent collagen fibrils interact to produce pyridinium cross-links. Degradation of collagen leads to two forms of stable pyridinium cross-links that are found in urine: (i) pyridinoline (pyr), a more abundant component derived from three hydroxylysine residues, and (ii) deoxypyridinoline (Dpyr), a less abundant component derived from one lysine and two hydroxylysine residues. Detection of an increased ratio of deoxypyridinoline (Dpyr) to pyridinoline (Pyr) cross-links in urine quantitated by high-performance liquid chromatography (HLPC) is a highly sensitive and specific test for *PLOD1*-kEDS [35, 36]. This biochemical test should be considered in case of a *PLOD1* VUS or a clinical diagnosis of *PLOD1*-kEDS.

## 5 Urine and serum analysis to screen for defects in GAG biosynthesis (spondylodysplastic and musculocontractural EDS)

Proteoglycans consist of a core protein with covalently attached glycosaminoglycan (GAG) chains, such as heparan sulfate (HS), chondroitin sulfate/dermatan sulfate (CS/DS), or keratan sulfate (KS). Genetic deficiencies of enzymes involved in HS and CS/DS biosynthesis lead to a wide range of inherited conditions including spondylodysplastic EDS (spEDS, bialleleic variants in *B3GALT6*, *B4GALT7* or *SCL39A13)* and musculocontractural EDS (mcEDS, biallelic recessive variants in *CHST14* and *DSE*). An elegant review by Syx et al. [37] describes the pathogenic mechanisms in detail but also highlights that urine and serum analyses may help diagnosing these conditions. Urinary disaccharide compositional analysis of CS/DS chains could provide a screening assay for mcEDS. Detection of increased amount of GAG-free bikunin core protein in serum by Western Blot analysis could function as a biomarker for spEDS [37]. These assays are not routinely available but could be considered in case of VUS or a strong clinical suspicion of spEDS or mcEDS and without abnormalities on genetic testing.

## 6 Conclusion

With increased application of NGS in the diagnosis of monogenic EDS, there is an increased probability to identify variants of unknown significance. Additionally, in some cases no genetic alteration is identified despite a strong clinical suspicion on a specific monogenic EDS type. To address these challenges in selected cases, functional and/or structural analyses that were used frequently in the past or have been recently developed can provide additional evidence that may confirm a clinical diagnosis of a monogenic EDS type. However, the options are limited, and there is a need for increased development of novel structural/functional studies for monogenic types of EDS. Ideally, genomic laboratories specialising in specific groups of disorders such as monogenic EDS could work closely with clinicians and researchers to develop, implement and offer these non-genetic investigations as the next line of investigation when NGS has not identified an underlying genetic cause despite a strong clinical suspicion on a specific monogenic EDS type. The level of evidence of specific structural/functional findings for application in the established DNA variant criteria remains to be determined.
